# Derivation of pb(II)-sensing *Escherichia coli* cell-based biosensors from arsenic responsive genetic systems

**DOI:** 10.1186/s13568-021-01329-y

**Published:** 2021-12-15

**Authors:** Yejin Lee, Yangwon Jeon, Guepil Jang, Youngdae Yoon

**Affiliations:** 1 Department of Environmental Health Science, Konkuk University, 05029 Seoul, Republic of Korea; 2 School of Biological Sciences and Technology, Chonnam National University, 61186 Gwangju, Republic of Korea

**Keywords:** Bacterial cell-based biosensors, Lead, ArsR, Protein engineering, Heavy metals

## Abstract

Heavy metal-responsive operons were used for the generation of *Escherichia coli* cell-based biosensors. The selectivity and specificity of the biosensors were determined based on the interaction between heavy metals and regulatory proteins; thereby, the modulating target selectivity of biosensors could be achieved by changing target sensing properties of regulatory proteins. The results of this study demonstrated that Pb(II)-sensing biosensors could be generated from an arsenic-responsive genetic system, which was originally used for arsenic-sensing biosensors. The amino acids around to As(III)-binding sites of ArsR were mutated and cysteine residues were relocated to modulate the metal selectivity. In addition, genes encoding metal ion-translocating P-type ATPases, such as *copA* and *zntA*, were deleted to enhance the specificity by increasing the intercellular levels of divalent metal ions. Based on the results, channel protein deleted *E. coli* cells harboring a pair of recombinant genes, engineered ArsR and *arsAp::egfp*, showed enhanced responses upon Pb exposure and could be used to quantify the amount of Pb(II) in artificially contaminated water and plants grown in media containing Pb(II). Although we focused on generating Pb(II)-specific biosensors in this study, the proposed strategy has a great potential for the generation of diverse heavy metal-sensing biosensors and risk assessment of heavy metals in environmental samples as well as in plants.

**Supplementary Information:**

The online version contains supplementary material available at 10.1186/s13568-021-01329-y.

## Introduction

Environmental pollutants including diverse organic chemicals, plastics, and heavy metal(loid)s produced in industrial processes are released into environmental systems as final reservoirs and are considered as potential threats to human health. Therefore, diverse pollutants are strictly regulated and regularly monitored to assess their risks. The monitoring and quantification of pollutants are typically carried out using analytical instruments. Although analytical instrument-based monitoring is very accurate and precise, it is expensive and requires time-consuming pretreatment processes because of the complex nature of the environmental matter. Furthermore, the risks of pollutants tend to be overestimated because the total amounts are measured instead of biologically active amounts. Therefore, alternative quantification methods must be established. Several quantification methods have been reported, which are based on different scientific technologies including biological, electrochemical, chemical, and nanotechnologies (Baruah and Dutta 2009; Hanrahan et al. 2004; Simonds et al. 1994; Zhou et al. 2017).

Among the new sensing techniques, bacterial cell-based biosensors have been actively investigated during the past few decades (Belkin 2003; Tecon and Van der Meer 2008). Most bacterial cell-based biosensors are based on stress-responsive genetic systems. For heavy metal sensing, metal-responsive genetic systems are used. Briefly, the recombinant genes constructed by fusing promoter regions and reporter genes encoding enzymes and fluorescent proteins are introduced to bacterial cells. The expression of the reporter genes is controlled by regulatory proteins in stress-responsive genetic systems. The level of interaction between the regulatory proteins and ligands results in the expression of reporter genes. Consequently, the ligands can be indirectly quantified based on the expression levels of the reporter genes. These biosensors are advantageous because they involve less expensive and less time-consuming processes; they can be used to quantify heavy metals in diverse matrixes compared with analytic instruments and to measure the biologically active amount of heavy metals, that is, the so-called bioavailability (Hynninen and Virta 2009; Yoon et al. 2016c). Although they have several advantages and huge potentials as alternative analytic tools, biosensors have not been widely used in practical applications. The reasons might include the lack of specificity and sensitivity in comparison with analytical instruments and the limited number of genetic systems compared with the number of heavy metals. In fact, stress-responsive genetic systems in living organisms generally have a broad specificity and sensitivity to ligands to efficiently respond because it could be attritional to possess all genetic systems toward stresses. Therefore, many research groups are investigating bacterial cell-based biosensors and make efforts to enhance the target specificity and selectivity based on genetic and protein engineering (Cerminati et al. 2015; Lee et al. 2020; Yagur-Kroll and Belkin 2014).

Considering the principles of bacterial cell-based biosensors, the specificity and selectivity depend on the level of the interaction between targets and regulatory proteins. Thus, the performance of biosensors can be enhanced by modulating regulatory proteins. Therefore, many researchers are focusing on the generation of new target-sensing biosensors and the enhancement of the sensing performances based on the genetic engineering of regulatory proteins in currently available biosensors such as GolS, ZntR, and ArsR (Cerminati et al. 2011; Kang et al. 2018; Lee et al. 2020). In addition, it has been reported that the heavy metal specificity/sensitivity of bacterial cell-based biosensors can be enhanced by interfering with the metal homeostasis in bacteria (Ibáñez et al. 2015; Yoon et al. 2018). The sensitivity to targets increases because deleting the genes related to the metal export results in the accumulation of target metal ions in bacterial cells.

In this study, a new strategy to generate new metal-sensing biosensors based on the above-mentioned procedure was introduced. We manipulated the target selectivity of ArsR, a regulatory protein controlling the arsenic responsive operon, by shifting it from As(III) to Pb(II). We also enhanced the sensitivity by deleting metal-exporting channels, such as CopA and ZntA, which are known to export metal ions from *Escherichia coli (E. coli*). The applicability of the new Pb(II)-sensing biosensors was evaluated by measuring the Pb(II) concentration in artificially contaminated water and plants grown in Pb(II)-bearing media.

## Materials and methods

### Materials

All heavy metal(loid)s tested in this study, including AsCl_3_, CdCl_2_, NiCl_2_, HgCl_2_, PbSO_4_, ZnCl_2_, and CuSO_4_, were purchased from Sigma-Aldrich (St. Louis, MO, USA). Metal(loid) solutions were prepared as 1 mM stock solutions by dissolving each metal(loid) salt in distilled water and ethanol. HotStar Taq and Pfu Turbo DNA polymerases were used for the DNA amplification and site-directed mutagenesis, respectively. Both were purchased from Qiagen. Restriction enzymes and T4 DNA ligase were purchased from Takara Korea Biomedical (Seoul, Korea). The primers used in this study were synthesized and purchased from Macrogen (Daejeon, Korea). A Quick & Easy *E. coli* Gene Deletion Kit (Gene Bridges, Heidelberg, Germany) was used to generate endogenous gene-deficient *E. coli* strains.

### Generation of *E. coli* cell-based biosensors

The *arsR*-encoding ArsR, a regulatory protein was amplified from the genomic DNA of *E. coli* by PCR. The gene was cloned into a pCDFDuet vector (Novagen) with BamHI/XhoI restriction enzyme sites. Mutations were introduced to ArsR by the site-directed mutagenesis using Pfu Turbo and the corresponding primers; the amino acid sequences of all engineered ArsR were confirmed by DNA sequencing. The plasmid-carrying *arsAp::egfp* was used as the sensing domain, which was constructed in our previous study (Yoon et al. 2016b). Gene deletion in *E. coli* was performed with the Quick & Easy *E. coli* Gene Deletion Kit following the manufacturer’s instructions, with minor modifications. Genes such as *zntA* and *copA*, encoding Zn(II)- and Cu(I)-translocating P-type ATPases, were replaced by kanamycin resistance genes, and the gene deletions were verified by PCR. The *E. coli* cell-based biosensors were generated by introducing a pair of plasmid-carrying *arsR* and *arsAp::egfp* to wild-type *E. coli* (BL21) and gene deficient strains.

### Genetic engineering of ArsR

The metal selectivity of *E. coli* cell-based biosensors changes upon the deletion of genes related to the metal homeostasis of the host cells. In addition, it has been reported that the selectivity of biosensors can be modulated by the genetic engineering of regulatory proteins (Lee et al. 2020; Yoon et al. 2018). The selectivity of *E. coli* cell-based biosensors based on an *ars*-operon system was determined based on the selectivity of ArsR. The results show that the selectivity of biosensors can be modulated by shifting the selectivity of ArsR. As previously reported, the arsenic-binding site of ArsR is a short helix region, amino acid numbers 30 to 39 sequenced as ELCVCDLCTA, and three cysteines are known to be the key residues for As recognition (Busenlehner et al. 2003; Shi et al. 1994). To evaluate the effects of cysteine residues, Cys 32, 34 and 37 were initially mutated to Ser, and then additional mutations were introduced. Briefly, deleted cysteines were relocated to Leu36, Thr38 or Ala39, and cysteines were added to Glu30 or Val33. In summary, ArsR W1-6 and YJ1-21 were generated and tested the metal selectivities along with WT ArsR. The amino acid sequences and the positions of cysteines for engineered ArsRs were listed in Table 1.

**Table 1:** List of engineered ArsR and metal(loid)s selectivity upon host *E. coli* strains

No	Name	Amino acid sequence (no. 30–40)	Cysteine location	Order of responding strength			References
				*E. coli-arsR*	*E. coli-arsR/copA*	*E. coli-arsR/zntA*	
1	WT	EL**C**V**C**DL**C**TA	Cys-32,34,37	As(9.8)	As(8.46)	As (7.38)>Pb (1.85)	Kim et al. (2020)
2	W4	EL**C**V**C**DLST**C**	Cys-32,34,39	As(5.7)>>Cd(1.95)	As(3.64)	Pb(3.03)>As(2.31)> Cd(1.73)	Lee et al. (2020)
3	W6	EL**C**V**C**D**C**STA	Cys-32,34,36	As(5.08)	As(4.23)	Pb(4.05)>Cd(2.56)>As(1.79)	Lee et al. (2020)
4	YJ6	**C**L**C**V**C**DLST**C**	Cys-30,32,34,39	As(3.34)	As(1.58)	Pb(2.31)>As(1.77)>Cd(1.53)	This study
5	YJ20	EL**C**V**C**DL**C**T**C**	Cys-32,34,37,39	As(4.54)	As(2.43)	As (2.71)>Pb (2.31)>Cd(1.3)	This study
6	W2	EL**C**V**C**DLSTA	Cys-32,34	As(11.34)	As(10.46)	As(9.98)>>Pb(2.06)	Lee et al. (2020)
7	W3	EL**C**V**C**DLS**C**A	Cys-32,34,38	As(18.40)	As(8.87)	As(9.13)>>Pb(1.67)>Cd(1.60)	Lee et al. (2020)
8	YJ2	**C**L**C**V**C**DLSTA	Cys-30,32,34	As(3.06)	As(2.21)	As (2.50)>Pb(1.57)	This study
9	YJ1	**C**LSV**C**DL**C**TA	Cys-30,34,37	–^‡^	–	–	This study
10	YJ4	E**C**SV**C**DLST**C**	Cys-31,34,39	–	–	–	This study
11	YJ5	**C**LSV**C**DLST**C**	Cys-30,34,39	–	–	–	This study
12	YJ11	EL**C**VS**C**LST**C**	Cys-32,35,39	–	–	–	This study
13	YJ14	EL**CC**SDLS**C**A	Cys-32,33,38	–	–	–	This study
14	YJ15	EL**CC**SDLST**C**	Cys-32,33,39	–	–	–	This study
15	YJ19	EL**C**VSDL**C**T**C**	Cys-32,37,39	–	–	–	This study
16	YJ21	ELSV**C**DLST**C**	Cys-34,39	–	–	–	This study
17	YJ3	**C**L**C**V**C**DL**C**TA	Cys-30,32,34,37	As(4.26)	As(2.16)	As(2.40)	This study
18	YJ7	**C**LSV**C**D**C**STA	Cys-30,34,36	–	–	–	This study
19	YJ8	**C**L**C**V**C**D**C**STA	Cys-30,32,34,36	–	–	–	This study
20	YJ12	EL**CCC**DLS**C**A	Cys-32,33,34,38	As (1.50)	–	Cd(1.60)>Pb(1.38)	This study
21	YJ13	EL**CCC**DLST**C**	Cys-32,33,34,39	As (2.41)	As(1.61)	Pb(1.43)>As(1.28)>Cd(1.26)	This study
22	YJ16	ELS**CC**DLS**C**A	Cys-33,34,38	–	–	–	This study
23	YJ17	ELS**CC**DLST**C**	Cys-33,34,39	–	–	–	This study
24	YJ18	EL**CCC**DLSTA	Cys-32,33,34	–	–	–	This study

^+^ Cysteines in amino acid sequences were indicated as bold letters and mutated amino acids were indicated with underlines

^‡^ Responding strengths toward heavy metal(loid) ions were indicated with induction coefficient values and the values under 1.25 were not shown

* The data were obtained by repeating more than 3 times and the standard deviations for induction coefficient values toward each metal(loid) was less than 10%

### Biosensor assay

The heavy metal(loid) selectivity of the biosensors to heavy metal(loid)s was tested using the following experimental conditions. The biosensor cells were grown overnight and then inoculated into fresh media. When the optical density of cells reached 0.4 (OD_600_), the cells were exposed to 5 µM of diverse heavy metal(loid) ions. After an exposure of 1 h, the expression levels of eGFP were measured using a fluorescence spectrophotometer (FC-2, Sinco, Korea) and excitation and emission wavelengths of 480 nm and 510 nm, respectively. Cells without heavy metal(loid) exposure were considered to be control samples, and the expression level of eGFP was represented as induction coefficient values (intensity of biosensors with metal exposure/intensity of the control). The *E. coli* cell-based sensors displaying target metal responses were further investigated to evaluate the metal specificity. To optimize the experimental conditions, the metal exposure, exposure duration, and concentration of heavy metal ions of the biosensor assay were determined under varying initial OD_600_ values of the cells.

### Plant growth and Pb(II) treatment

*Arabidopsis thaliana* ecotype Columbia (Col-0) was used as a model plant to quantify the Pb(II) accumulation in plants. Seeds were sterilized using 70% ethanol and plated on half-strength Murashige and Skoog (1/2 MS) agar media containing 0, 250, 500, and 750 µM of Pb(II). The plates were placed in the dark for 3 d for vernalization, and plants were grown in a growth chamber with a 16 h/8 h (light/dark) light regime at 23 °C for 14 d. Images of the plants were taken to verify the inhibitory effects of Pb(II), and the plants were harvested to quantify the Pb(II) accumulation.

### Quantification of Pb(II) using biosensors

#### Artificially contaminated water

To verify the application of Pb(II) biosensors, artificially contaminated water samples were prepared by spiking metal-free water with Pb(II). The final concentrations were set to 100, 250, 400, and 500 µM. Standard curves for the quantification were constructed by fitting induction coefficient values against 0 to 5 µM ranges of Pb(II). The contaminated water samples were applied to biosensor cells after dilution, and the induction coefficient values were determined by using the above-mentioned procedures. Subsequently, the Pb(II) concentration of the biosensors was calculated using the linear regression fit obtained from the standard curves.

#### Plants exposed to Pb(II)

To quantify the Pb(II) accumulation in plants, dried plants grown in Pb(II)-bearing media were grinded and directly applied to *E. coli* cell-based biosensors, that is, plant samples with a dried weight (DW) of 10 mg were added to 2.5 mL biosensor cells. The induction coefficient values were determined after an incubation of 2 h. The Pb(II) accumulation in the plants was quantified by using the linear regression fit obtained from the standard curves. The amount of Pb(II) in plants was represented as µg/g DW.

## Results

### Effects of ***copA*** and ***zntA*** deletion on the metal selectivity of WT ArsR

**Fig. 1:**
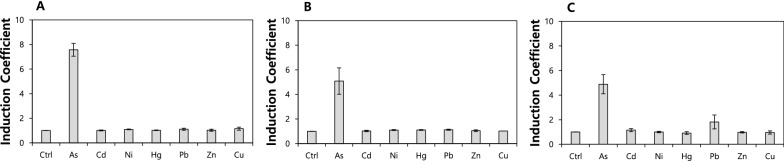
Effects of *copA* and *zntA* deletions in *E. coli* on the metal selectivity of ArsR WT. The *E. coli* cell-based biosensors with ArsR WT as regulatory proteins were exposed to 5 µM metal(loid) ions including As(III), Cd(II), Ni(II), Hg(II), Pb(II), Zn(II), and Cu(II). The expression levels of eGFP upon metal(loid) exposure are represented as induction coefficient values. **A** *E. coli-arsR*, **B** *E. coli-arsR/copA*, and **C** *E. coli-arsR/zntA* were used as host cells for the biosensors

In this study, *E. coli* cell-based biosensors were generated by inserting a pair of plasmids, pArsAp-eGFP and pCDF-ArsR, into *E. coli* BL21-*arsR*, -*arsR/copA*, and -*arsR/zntA* and by exposing them to 5 µM metal(loid) ions, including As(III), Cd(II), Ni(II), Hg(II), Pb(II), Zn(II), and Cu(II). Although it was not tested the toxic effects of each metal(loid) on growth of *E. coli*, some metal(loid)s such as As(III) and Hg(II) showed inhibitory effects over 10 µM of exposure. In this reason, 5 µM was selected to test selectivity of biosensors to metal(loid)s without inhibition of *E. coli* growth. After an exposure of 2 h, the responses were determined by measuring the expression levels of eGFP. The results were represented as induction coefficient values (Fig. 1). As shown in Fig. 1A, WT ArsR in *E. coli-arsR* responds to As(III), which is consistent with previous reports (Yoon et al. 2016a). In the case of *E. coli-arsR/copA*, the change was insignificant, indicating that the effect of deleting *copA* on the metal specificity is small (Fig. 1B). On the other hand, *E. coli* cell-based biosensors display a stronger response to Pb(II) after the deletion of *zntA* (Fig. 1C). Note that the gene related to the translocation of divalent metal ions, *zntA*, which encodes Zn(II)-translocating P-type ATPase, enhances the responses of biosensors to Pb(II). Although it remains unclear why only the Pb(II) response is enhanced, the results show that the specificity and selectivity of biosensors could be modulated by interfering with the metal ion homeostasis in *E. coli* cells (Kang et al. 2018).

### Effects of ArsR engineering on the heavy metal(loid) selectivity

**Fig. 2:**
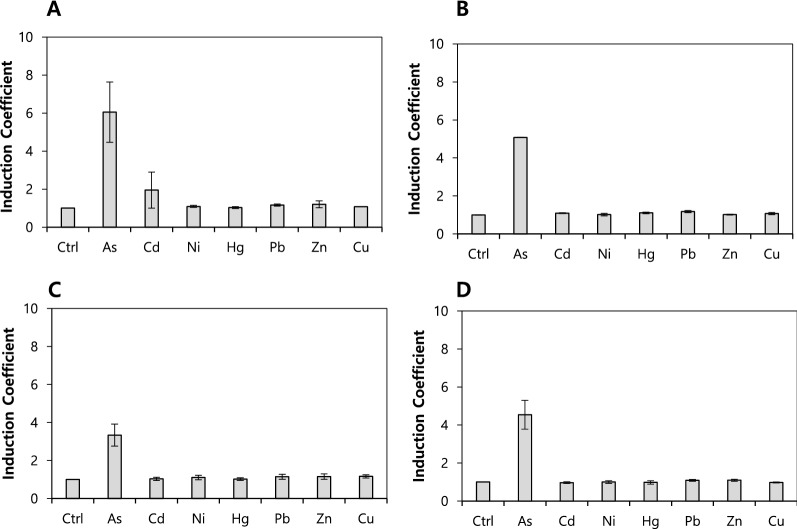
Effect of the genetic engineering of ArsRs on the metal selectivity of biosensors based on *E. coli-arsR*. *E. coli-arsR* harboring pArsAp-eGFP and engineered ArsRs including ArsR W4 (**A**), ArsR W6 (**B**), ArsR YJ6 (**C**), and ArsR YJ20 (**D**) exposed to 5 µM of metal(loid) ions for 2 h

Biosensors based on *E. coli-arsR* were generated by using engineered ArsRs and pArsAp-eGFPA as sensing and reporter domains, respectively. A series of biosensors was exposed to 5 µM heavy metal(loid)s including As(III), Cd(II), Ni(II), Hg(II), Pb(II), Zn(II), and Cu(II) and the expression of eGFP was measured after an exposure of 2 h. The responses to heavy metal(loid)s were measured as eGFP intensity and converted into induction coefficient values (eGFP intensity with metal exposure/eGFP intensity without exposure). The results are summarized in Table 1. In concordance with our previous report, the biosensor with wild-type ArsR has a strong selectivity and specificity with respect to As(III) (Lee et al. 2020; Yoon et al. 2016a). However, the response to metal ions diminishes and is lost after the deletion of Cys32 or Cys34 with Val33Cys in ArsR, corresponding to ArsR numbers 9 to 16 in Table 1. They showed induction coefficient values below 1.25 toward tested heavy metal(loid) ions. It was determined that two cysteine residues (32 and 34) are the key amino acids for the recognition of metal ions. On the other hand, engineered ArsR with Cys32/Cys34 or with cysteines on Thr38 instead of Cys37 showed a stronger response to As(III) than WT ArsR without changes in the metal selectivity. Insignificant changes of the metal selectivity were observed for engineered ArsRs with 3 and 4 cysteines, that is, numbers 17 to 24 in Table 1. Although several ArsRs responded to As(III), the background eGFP intensity increased with and without metal exposure. This implies that the mutations cause conformational changes, which result in inactive ArsR as a repressor for *ars*-operon. The metal selectivity of the biosensors with ArsR W4, W6, YJ6, and YJ20 are shown in Fig. 2. Although the selected mutants have similar responses to metal(loid) ions as ArsR WT, the response to As(III) decreased and was very weak, less than 1.25 of induction coefficient values toward divalent metal ions.

### Effects of genetic engineering of ArsR and host cells on the metal selectivity

As shown above, the As(III) responses of biosensors decreased due to the engineering of ArsR in *E. coli-arsR*. The effects of metal-exporting channels, that is, *copA* and *zntA*, which are known to encode metal ion-translocating P-type ATPases, on the metal specificity were evaluated using *arsR*/*copA*- and *arsR*/*zntA*-deficient *E. coli* strains named *E.coli-arsR/copA* and *E. coil-arsR/zntA*, respectively. By using those strains as host cells, biosensors were generated by inserting *arsAp::egfp* and engineered ArsRs; the changes of the metal selectivity were determined under the experimental conditions specified above. Although the induction coefficients slightly change, the *copA* deletion has a significant effect on the metal selectivity compared with that of *E. coli-arsR* (Table 1).

**Fig. 3:**
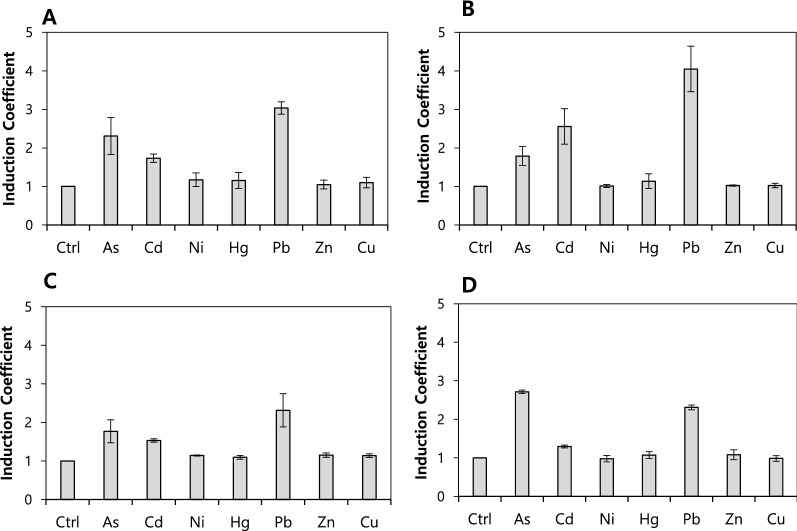
Metal selectivity of biosensors based on *E. coli-arsR/zntA* upon engineered ArsRs. *E. coli-arsR/zntA* harboring pArsAp-eGFP and engineered ArsRs including ArsR W4 (**A**), ArsR W6 (**B**), ArsR YJ6 (**C**), and ArsR YJ20 (**D**) exposed to 5 µM of metal(loid) ions for 2 h

On the other hand, the biosensors show different responses to divalent metal ions after the deletion of *zntA* (Table 1; Fig. 3). First, the responses of the biosensors to divalent metal ions, such as Cd(II) and Pb(II), significantly enhanced compared with biosensors based on *E. coli-arsR*. Second, the responses of engineered ArsR to As(III) decreased compared with WT ArsR (Figs. 1C and 3). In the cases of engineered ArsR W4 and W6, the responses to As(III) and Pb(II) decreased two- and four-fold, respectively. The ArsR W6 showed a two times stronger response to Pb(II) compared with As(III), even if it significantly responded to Cd(II) (Fig. 3A). The ArsR W4 showed a 1.5 times stronger response to Pb(II) compared with As(III) and responded less to Cd(II) (Fig. 3B). The ArsR YJ6 and YJ20 also showed enhanced responses to Pb(II) and weakened responses to As(III) (Fig. 3C and D). Based on these results, it can be speculated that the mutation of ArsR changes the target selectivity, and the deletion of *zntA* enhances the target specificity based on the accumulation of divalent metal ions in the cells. The results also reveal that ZntA is related to the translocations of Zn(II), Cd(II), and Pb(II) ions.

### Pb(II)-sensing *E. coli* cell-based biosensors

**Fig. 4:**
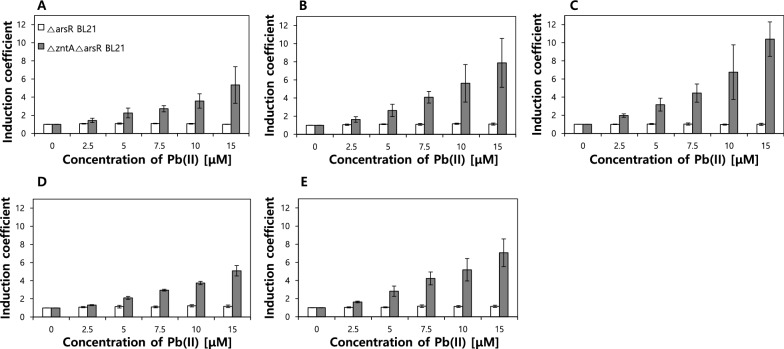
Concentration-dependent responses of biosensors to Pb(II). The responses of biosensors using *E. coli-arsR* and *E. coli-arsR/zntA* as host cells to Pb(II) were compared upon regulatory proteins. The concentration of Pb(II) ranged from 0 to 15 µM. The responses are indicated as induction coefficient values. **A** ArsR WT; **B** ArsR W4; **C **ArsR W6; **D** ArsR YJ6; and **E** ArsR YJ20

Although biosensors including a combination of engineered ArsRs and *zntA-*deficient *E. coli* show broad responses to As(III), Cd(II), and Pb(II), the selected engineered ArsRs, that is, W6, W4, YJ20, and YJ6, display stronger responses to Pb(II) than As(III) (Fig. 3). Therefore, they were further investigated to evaluate their potentials as Pb(II)-sensing biosensors. The biosensors were exposed to a Pb(II) concentration ranging from 0 to 15 µM, and the expression of eGFP induced by Pb(II) was measured after 2 h at excitation and emission wavelengths of 480 nm and 510 nm, respectively. The responses were represented as induction coefficient values and the relationship. In *E. coli-arsR/zntA*, all tested biosensors showed increased signals with increasing concentration (Fig. 4). The ArsRs W6, W4, and YJ20 showed stronger responses to Pb(II) than ArsR WT, and ArsR YJ6 displayed similar responses. Because they still responded to As(III) and Cd(II), they cannot be regarded as Pb(II)-specific biosensors. However, it can be concluded that the Pb(II) specificity was enhanced while the As(III) specificity was diminished due to the genetic engineering of both regulatory proteins and host cells.

### Pb quantification in artificially contaminated water

As described above, in addition to responses to As(III) and Cd(II), a Pb(II)-sensing capability was observed by genetic engineering on ArsR and host cells. Thus, it was necessary to evaluate the capabilities of the new Pb(II)-sensing biosensors. With respect to amino acid sequences at the metal binding sites of ArsRs, W4 and W6 have three cysteines, whereas YJ6 and YJ20 have four cysteines (Table 1). Among 4 candidates, biosensors with ArsR W6 and YJ20 were selected for the Pb(II) quantification because they show stronger Pb(II) responses based on the presence of three and four cysteines. The performance of the biosensors was analyzed to determine the Pb(II) concentration in artificially contaminated water samples that were prepared by spiking metal-free water with a known concentration of Pb(II). The Pb(II)-spiked water samples were applied to biosensors based on *E. coli-arsR/zntA* with ArsR W6 and ArsR YJ20 with diverse dilutions. The induction coefficient values of the contaminated water samples were measured, and the concentration was calculated by using the equations obtained from the fitting of the standard curves (Additional file 1: Figure S1). The arbitrary unit of biosensors without Pb(II) exposure was 300 to 350, and 0.5 µM of Pb(II) exposure showed about 1.3 times higher. The detection limit was calculated as about 0.15 of induction coefficient based on standard deviation of 0.5 µM of Pb(II) exposure and slope of standard curves. The Pb(II) concentrations of the artificially contaminated water samples was listed in Table 2. Compared with originally spiked Pb(II), the accuracy of the Pb(II) quantification using both biosensors varied from 95 to 99% with an error range of 3.5–12.5%.

**Table 2:** Quantification of Pb in artificially contaminated water samples using genetically engineered *E. coli* biosensors

Spiked conc. [µM]	*E. coli-arsR/zntA* with W6		*E. coli-arsR/zntA* with YJ20	
	Determined conc. [µM]	Accuracy ^+^ (%)	Determined conc. [µM]	Accuracy (%)
100	101.3 ± 11.1	99.0	96.3 ± 10.1	96.3
250	257.9 ± 14.6	96.9	254.3 ± 31.5	98.3
400	416.5 ± 31.0	96.0	381.4 ± 38.9	95.2
500	524.8 ± 39.3	95.3	491.6 ± 18.6	98.3

^+^ Accuracy was calculated by the coverage of Pb quantification over originally spiked concentrations

### Quantification of Pb(II) in plants

To prepare Pb(II)-bearing plant samples, *Arabidopsis thaliana* was grown in MS media containing different Pb(II) concentrations ranging from 0 to 1000 µM. After an incubation of 14 d with 16 h of light/8 h of darkness at 23 ℃, images of the plants on each plate were taken, and the plants were harvested and dried. At a Pb(II) concentration of 250 µM, seed germination and growth were observed. Above a concentration 750 µM, germination did not occur (Additional file 1: Figure S2). The inhibition of seed germination and development of lead toxicity have previously been investigated by many research groups (Kabir et al. 2008; Li et al. 2005; Mahmood et al. 2007). Most of the researchers focused on the correlation between the exterior concentration of Pb(II) and physiological variation of plants. However, the correlation between the Pb(II) accumulation and physiological changes must be determined.

To determine the Pb(II) accumulation in the plants, the plant samples were grinded and added to *E. coli* biosensors. After an exposure of 2 h, the expression levels of eGFP were measured, and the amount of accumulated Pb(II) was calculated by using the equations obtained from the fitting of standard curves. The concentration of Pb(II) accumulated in plants grown in MS media containing 250 and 500 µM Pb(II) was determined to be 352.22 ± 114.02 and 1263.77 ± 330.13 µg/g DW, respectively (Additional file 1: Table S1). In this regard, it was important to quantify accumulated Pb(II) to address correlation between adverse effects on the physiological properties of plants and Pb(II) exposure and to achieve more accurate risk assessments.

## Discussion

Bacterial cell-based biosensors have been widely investigated as alternative quantification tools for environmental monitoring because of their simplicity, low cost, rapid analysis, and bioavailability assessment. Most bacterial cell-based biosensors have common genetic systems consisting of sensing and reporter domains that recognize targets and indicate responses, respectively. The target selectivity of *E. coli* cell-based biosensors can be determined by the selectivity of regulatory proteins in the genetic systems. It has been suggested that the target selectivity can be shifted by modulating the target selectivity of regulatory proteins. In addition, the results of previous studies revealed that the deletion of genes related to endogenous metal ion homeostasis in host cells leads to changes in the target selectivity and specificity (Kim et al. 2020; Yoon et al. 2018).

In this study, *copA*- and *zntA*-deficient *E. coli* strains were used as host cells for biosensors to verify the effects of genes related to metal homeostasis. Metal ion-translocating P-type ATPases in *E. coli*, such as CopA and ZntA, are known to control the levels of heavy metal(loid)s in cells (Rensing et al. 1997, 2000; Sharma et al. 2000). As shown in Fig. 1, the As(III) selectivity of the biosensors containing ArsR WT as regulatory proteins changed after the deletion of *zntA* while the deletion of *copA* had no effect on the target selectivity. Even if ArsR WT was used as a regulatory protein in each biosensor, Pb(II) responses were observed after *zntA* deletion. It has been suggested that ZntA, that is, Zn(II)-translocating P-type ATPase, is related to Pb(II) translocation and the deletion of *zntA* causes the accumulation of Pb(II) in the cells. The concentrations of other divalent metal ions, such as Cd(II), Ni(II), and Zn(II), in the cells might be increased, but they cannot activate ArsR.

As described above, the target selectivity was determined based on the selectivity of regulatory proteins in bacterial cell-based biosensors. Because *E. coli* cell-based biosensors based on an *ars*-operon system were regulated by ArsR, the As(III) binding sites were mutated to modulate the metal selectivity. As shown in Table 1, cysteine residues at the binding sites were mutated and relocated to generate engineered ArsRs. The target selectivity of ArsRs was tested in *E. coli-arsR*, *E. coli-arsR/copA*, and *E. coli-arsR/zntA* with pArsAp-eGFP. The ArsRs with both Cys32 and Cys34 show the strongest response to As(III) and the metal response in ArsRs without Cys32 or Cys34 is lost. On the other hand, several engineered ArsRs, that is, numbers 17 to 24 in Table 1, lose their repressor ability, resulting in a strong eGFP expression, even without metal exposure. This could be explained by conformational changes upon mutation, which affected the DNA binding of ArsR. On the other hand, the engineered ArsR W4, W6, YJ6 and YJ20 showed decreased As(III) and increased Pb(II) responses in *zntA*-deficient *E. coli*. Additionally, those biosensors showed concentration-dependent responses to Pb(II) as shown in Fig. 4. So far, lead-responsive operon, *pbr*, was the only genetic system for lead detecting bacterial cell-based biosensors (Jia et al. 2018; Wei et al. 2014). In this regard, it was notable to generate Pb(II) responding biosensors from arsenic-responsive operon although it was not highly specific to Pb(II).

Despite many advantageous aspects, bacterial cell-based biosensors were not applied widely to environmental systems. Thus, it was pivotal to elucidate applicable potentials of biosensors for real environmental systems. In this study, new biosensors were used for the quantification of Pb(II) in artificially contaminated water samples and plants grown in contaminated media. The new biosensors yielded an accuracy above 95% with respect to the quantification of Pb(II) in artificially contaminated water samples (Table 2). Although it was not environmental samples collected in the field, it showed the potentials of new biosensors for quantifying Pb(II) in water systems. In addition, the new Pb(II) biosensors were applied to plants grown in Pb(II)-exposed media to quantify the amount of accumulated Pb(II), which was converted to µg/g DW. The plants exposed to Pb(II) concentrations of 250 and 500 µM exhibit growth inhibition and contain 352 and 1263 µg/g Pb (II) DW, respectively (Additional file 1: Table S1 and Figure S2). Although the results were not validated based on the comparison with the instrumental analysis in this study, the biosensors are invaluable simple tools that can be used to quantify the Pb(II) accumulation in plants. The effects of toxic materials on plants have been investigated to determine the correlation between the exterior concentration and physiological properties. However, it was insufficient to assess risks of toxic materials because physiological properties such as intake rates and resistance for toxic materials were varied upon plant species. Therefore, the quantification of the accumulated amount could provide more accurate risk assessment for plants rather than exterior amounts.

In this study, we demonstrated the approaches to modulate target selectivity and specificity of bacterial cell-based biosensors. The metal selectivity of the biosensors was shifted from As(III) to Pb(II) by genetic engineering on regulatory protein and the specificity was enhanced by interfering metal homeostasis in host cells. However, it was still necessary to improve performance of biosensors for practical application because of broad selectivity with respect to Pb(II), As(III) and Cd(II). Even if the applicability of biosensors was evaluated by quantifying Pb(II) in water and plant samples, the results would be exaggerated by the presence of As(III) or Cd(II). In this regard, it was critical to achieve target selectivity and specificity of biosensors to achieve accurate risk assessment. Nonetheless, it would be noticed that the strategy demonstrated here has a great potential with respect to the generation of diverse heavy metal-sensing biosensors based on existing genetic systems and assessment of the risks of heavy metals in environmental systems.

## Supplementary Information

**Additional file 1.** Additional table and figures.

## Data Availability

All data generated or analyzed during this study are included in this manuscript.
