# Supplementary material for: Derivation of pb(II)-sensing Escherichia coli cell-based biosensors from arsenic responsive genetic systems
Source: AMB Express. 2021 Dec 15;11:169. doi: 10.1186/s13568-021-01329-y (PMC8674403; doi:10.1186/s13568-021-01329-y)

Applied Microbiology and Biotechnology Express

Supplementary data for:

Derivation of Pb(II)-sensing *Escherichia coli* cell-based biosensors from arsenic responsive genetic systems

Yejin Lee[1], Yangwon Jeon[1], Guepil Jang[2], Youngdae Yoon[1,*]

[1]Department of Environmental Health Science, Konkuk University, Seoul 05029, Republic of Korea
[2]School of Biological Sciences and Technology, Chonnam National University, Gwangju 61186, Republic of Korea

***Corresponding author: Youngdae Yoon,** Konkuk University, Korea**;** yyoon21@gmail.com

Supplementary Table 1. Quantification of accumulated Pb(II) in Arabidopsis thaliana grown at Pb(II) containing MS media

Pb(II) in MS plates [μM]	Pb(II) determined by biosensor [μM]	Accumulated Pb(II) in plants [μg/g of DW]
250	2.04±0.66	352.22±114.02
500	7.33±1.91	1263.77±330.13

Supplementary Figure 1. Standard curves for determining Pb in artificially contaminated water samples obtained from *E. coli-arsR/zntA* with ArsR W6 (A) and with ArsR YJ20 (B).

(A)

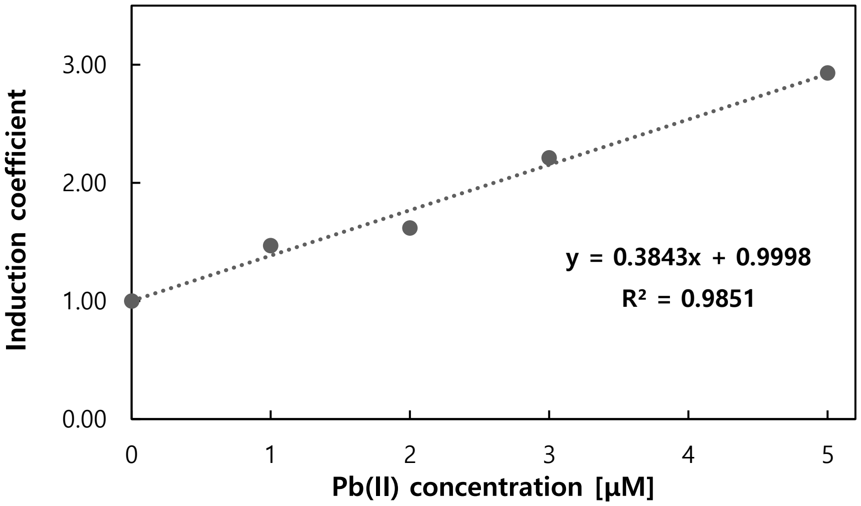

(B)

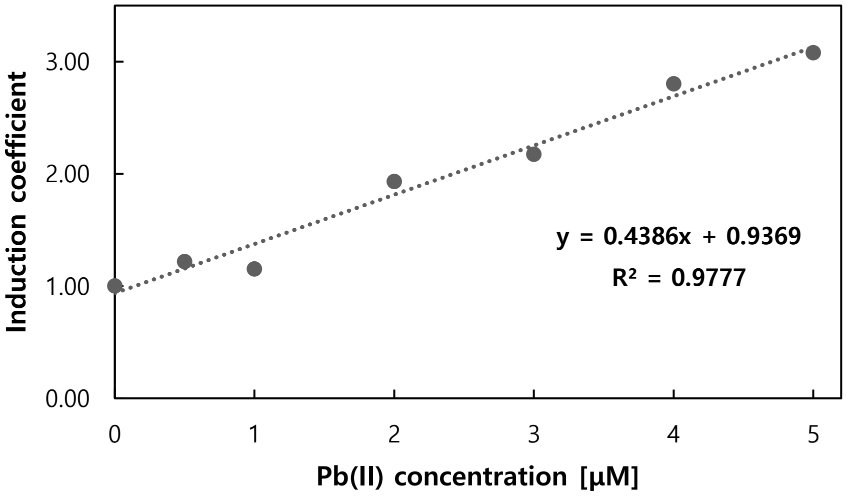

Supplementary figure 2. Inhibitory effects of Pb on growth of *Arabidopsis thaliana*.

Plants were grown on half-strength MS agar plates containing 0 μM (A), 250 μM (B), 500 μM (C) and 750 μM (D) of Pb. Images of plates were taken after 2 weeks incubations.

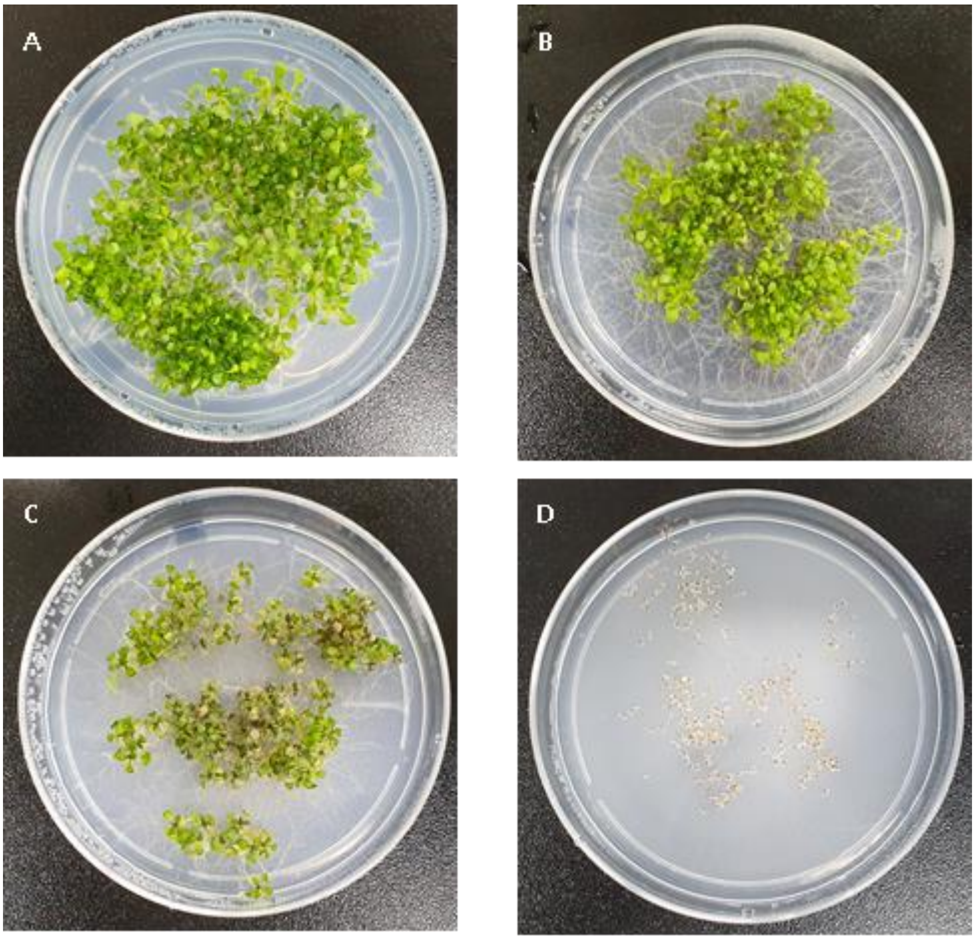

Supplement: Supplementary file 1 — Additional file 1. Additional table and figures. [file 13568_2021_1329_MOESM1_ESM.pdf]
